# Life and Leisure Activities following Stroke or Transient Ischaemic Attack (TIA): An Observational, Multi-Centre, 6-Month Follow-Up Study

**DOI:** 10.3390/ijerph192113848

**Published:** 2022-10-25

**Authors:** Matthew J. Reeves, Clare Thetford, Naoimh McMahon, Denise Forshaw, Chris Brown, Miland Joshi, Caroline Watkins

**Affiliations:** 1 UCLan Research Centre for Sport, Physical Activity & Performance, University of Central Lancashire, Preston PR1 2HE, UK; 2 Lancashire Institute for Global Health and Wellbeing, University of Central Lancashire, Preston PR1 2HE, UK; 3 Stroke Research Team, University of Central Lancashire, Preston PR1 2HE, UK; 4 Division of Health Research, University of Lancaster, Lancaster LA1 4YW, UK; 5 Lancashire Clinical Trials Unit, University of Central Lancashire, Preston PR1 2HE, UK

**Keywords:** stroke, TIA, leisure, physical activity, rehabilitation, sport

## Abstract

Objective: To examine changes in leisure participation following stroke/transient ischaemic attack (TIA) and explore its relationship to modifiable and non-modifiable participant characteristics. Design: An observational study design with self-report questionnaires collected at two time points (baseline and 6-months). Setting: The study was conducted across 21 hospital sites in England, Wales, and Northern Ireland. Participants: Participants were aged 18+ and had experienced a first or recurrent stroke or TIA and had a post-stroke/TIA modified Rankin score (mRS) of ≤3. Procedure: Research practitioners at each site approached potential participants. Individuals who agreed to participate completed a baseline questionnaire whilst an inpatient or at a first post-stroke/TIA clinic appointment. A follow-up questionnaire was posted to participants with a freepost return envelope. Two questionnaires were developed that collected demographic information, pre-stroke/TIA mRS, social circumstances (e.g., employment situation) and incorporated the shortened Nottingham Leisure Questionnaire (sNLQ). Results: The study recruited eligible participants (N = 3295); 2000 participants returned questionnaires at follow-up. Data showed three participant variables were significant predictors of engagement in leisure activities post-stroke/TIA: age, sex, and deprivation decile. There was an overall decline in the number and variety of leisure activities, with an average loss of 2.2 activities following stroke/TIA. Only one activity, “exercise/fitness” saw an increase in engagement from baseline to follow-up; watching TV remained stable, whilst participation in all other activities reduced between 10% and 40% with an average activity engagement reduction of 22%. Conclusions: Some groups experienced a greater reduction in activities than others—notably older participants, female participants, and those living in a low socioeconomic area. Registration: researchregistry4607. Strengths and limitations of this study: 1. This is the largest-ever study to survey life and leisure activity engagement following stroke/TIA. 2. Survey responses were self-reported retrospectively and, therefore, may have been misreported, or misremembered. 3. Despite the large cohort, there were few participants, and so respondents, from ethnic minority groups.

## 1. Introduction

Stroke is a major cause of death and disability worldwide [1,2]. It is estimated that there are over 13.7 million new cases of stroke annually worldwide and one in four people over the age of 25 will suffer stroke in their lifetime [3]. Stroke can lead to a range of complex and permanent effects, including problems with movement, cognition, vision, communication, and psychological state [4,5,6]. Such effects can have a profound impact on quality of life, with around two thirds of stroke survivors reporting reduced participation in activities that they valued pre-stroke [7,8,9]. Even in instances of mild stroke, where motor function or the ability to perform basic activities of daily living (ADL) are marginally compromised, around one-third of people report not having regained previous levels of participation in daily activities (e.g., driving, employment, relationships, and leisure) at 6-months post-stroke [10].

Within stroke recovery/rehabilitation there is a strong focus upon reducing physical dependence on others and, as such, studies in this area have focussed on performing ADLs [11], which is reflective of the priority given to physical recovery in current clinical practice [11,12]. However, it fails to consider the wider psycho-social benefits that might be derived from leisure and physical activities. A randomised controlled trial of leisure therapy versus usual treatment following stroke found no clear beneficial effect upon mood state at 6- or 12-month follow-up [12]. This study, however, excluded potential participants against strict criteria, creating a select sample and size that was not systematically identified, and did not find firm evidence of efficacy of the intervention used. A recent systematic review evaluating the use of leisure therapy in stroke rehabilitation [13] concluded that the overall quality of studies did not reach the highest level of methodological rigour and that further controlled research is required to inform and develop evidence-based guidelines for leisure-based rehabilitation.

Similarly, there has not been sufficiently purposeful consideration of the value and impacts of leisure activities when exploring long-term unmet needs following stroke. Indeed, it is suggested that leisure provides meaning and purpose during recovery [14,15]. However, research and practice remain focussed on recovery of physical function rather than on enabling a return to previously valued social and leisure activities [15,16,17].

This gap in stroke rehabilitation merits attention for several reasons. Firstly, the demographic of stroke survivors consists of a high number of older adults of retirement age [18], who may be particularly vulnerable to social isolation after stroke due to not having social contact through employment. Additionally, one in three stroke survivors will experience post-stroke depression [19]; whilst engagement in valued leisure activities has been shown to be positively associated with improvement in emotional well-being after stroke [20]. Further, a return to outdoor activities has been identified to be of interest for people after stroke [21], yet nearly half of stroke survivors experience outdoor mobility restrictions [22]. There is a growing body of evidence demonstrating the beneficial effects of exercise and leisure in natural, as opposed to synthetic or clinical environments [23,24,25]. Although limited, evidence from activities including golf [26] and cycling using electric bicycles [27] suggest that social engagement, physical health, and independence can be positively affected. However, further research is needed to examine stroke-specific barriers and enablers to participation in outdoor leisure activities.

There are no large-scale studies that we are aware of, that have sought to understand the leisure practices of people following stroke/transient ischaemic attack (TIA) and at 6-months post-event. Such data are critical in understanding the implications of leisure changes following stroke as this can affect the development of future intervention or support programmes that can more effectively use the mechanisms of social prescribing to maintain engagement in leisure activities. The aim of the current study was to examine changes in leisure participation following stroke/TIA and explore differences by participant characteristics.

## 2. Methods

### 2.1. Design

This was an observational study with data collected using self-report questionnaires at baseline and 6-month follow-up. The study protocol was approved by the Wales Research Ethics Committee 5, Bangor (ID: 17/WA/0336) and was registered online (https://www.researchregistry.com; 4607). The data that support the findings of this study are available from the UCLanData repository (https://uclandata.uclan.ac.uk/). Written informed consent, or assent from consultees, was obtained from all participants.

### 2.2. Setting

This study was conducted across 21 hospital sites in England, Wales, and Northern Ireland.

### 2.3. Participants

Participants were recruited using the following inclusion criteria: (1) adults aged 18 or over; (2) clinical diagnosis of new first or recurrent stroke or TIA; (3) a pre-stroke mRS of ≤3 (An mRS score of; 0 equates to no symptoms; 1 indicates no significant disability despite symptoms and ability to perform all usual activities; 2 signifies slight disability and an inability to perform all previous activities; and 3 denotes moderate disability where some assistance with activities is required but are able to walk without assistance. All mRS scores over 3 suggest it would be unlikely for individuals to engage in most leisure activities due to the severity of their stroke/TIA-related disability.); (4) the capacity to consent, or a suitable consultee able to provide consent; and (5) can communicate in English or has a suitable consultee who can assist in completing the questionnaire. Possible participants were excluded if their clinical care team identified them as being in the last days or weeks of their life. The mRS was selected because it is easy for participants to rate themselves, and it also is a frequently used stratification and outcome assessment in both observational and interventional studies.

### 2.4. Sampling and Sample Size

Participating sites were asked to recruit the first 15 people that consented to participation for each month of recruitment and were encouraged to do so in a 2:1 ratio of stroke to TIA to ensure we retained the study focus on leisure after stroke, without losing sight of difficulties after TIA. Each site was given recruitment targets for each month over a 12-month period (between December 2017 to April 2019). The decision to recruit at a 2:1 ratio was pragmatic and in recognition of different sites’ operation and services. For example, some sites encompassed TIA within the ward setting of acute stroke whereas others operated TIA-specific clinics.

A 12-month recruitment period was considered appropriate to ensure a spread of recruitment over the study period. It accounted for seasonal variation that might affect access and willingness to undertake outdoor leisure activities; it also allowed factors affecting equity of access to leisure facilities (i.e., city versus rural sites) to be addressed. Where sites were unlikely to hit their recruitment target, they contacted the Lancashire Clinical Trials Unit (LCTU) who requested other sites to recruit additional participants.

We aimed to recruit 3240 participants to the study; based upon an estimated attrition rate for the return of follow-up questionnaires of 40%—it was anticipated this would result in an estimated 1944 respondents completing both baseline and follow-up questionnaires. This figure was calculated to ensure the sample would achieve the required minimum sample sizes to conduct subgroup analyses for demographic variables, disability, and social circumstances using the shortened Nottingham Leisure Questionnaire (sNLQ). We assumed a between-participant standard deviation (SD) in sNLQ of 6.46; this is based on the 6-month SD of sNLQ [12], pooled (7.56) with a moderately large correlation between baseline and outcome of 0.635 or doubling the reported test–retest SD of 3.23 [28]. When examining patient characteristics, we aimed to obtain a minimum of 300 complete records to be able to detect a fall in the average sNLQ of about 0.4 between baseline and follow-up responses.

### 2.5. Procedure

Research practitioners based on site from the 21 sites involved approached potential participants, or a suitable consultee (i.e., carer/friend) about the study whilst an inpatient, or at a first post-stroke/TIA clinic appointment. Potential participants were provided with the study information sheet and had the opportunity to ask any questions they had about the study. Recruitment, wherever possible, was consecutive and monitored using screening logs.

Participants who consented to involvement in the study completed a baseline questionnaire whilst in hospital or TIA clinic. Follow-up questionnaires were posted to participants by the Lancashire Clinical Trials Unit (LCTU) 6-months after the date of participants’ stroke/TIA. A freepost envelope was provided to enable participants to return questionnaires directly to the LCTU. Completion reminders were not sent to participants for cost-related reasons. Figure one illustrates participant recruitment, questionnaire completion, exclusions, and 6-month follow-up figures.

### 2.6. Questionnaires

Two questionnaires were developed for use in the study. The baseline questionnaire was used to collect demographic information (i.e., age, sex, ethnicity), pre-stroke/TIA mRS, and information on social circumstances (for example, where people were living and with whom, employment, primary mode of transport).

We used a validated tool, the sNLQ [28,29], to collect information on pre-stroke/TIA leisure participation. The tool lists 30 leisure activities and respondents are asked to indicate, using a three-point Likert scale (i.e., regularly, occasionally, never), how often they have participated in each activity over the past few weeks.

The 6-month follow-up questionnaire asked about self-assessed disability, social circumstances, and included a repeat sNLQ, along with additional open-ended questions regarding perceived barriers and enablers to engaging in leisure activities; and two questions related to mood and fatigue.

### 2.7. Data Analysis

All demographic, diagnostic, and sNLQ data were entered into Stata 15/16 where they were screened for missing values, input accuracy, and analysed descriptively by LCTU. Engagement (regularly or occasionally) in leisure activities at baseline and 6-month follow-up was tabulated and percentage changes calculated for each activity. Paired *t*-tests were performed to investigate differences in the number of activities and total sNLQ scores between pre- and post-stroke/TIA event, and 95% confidence intervals were constructed for the difference. sNLQ scores/counts were calculated using the assumption that if a participant had completed at least one item within sNLQ at a specific time-point then any missing items would be taken as “never” undertaken at that time point. Sensitivity analysis for the paired t-tests were conducted using another assumption: that if there was a missing response to an individual item within the sNLQ that the respective count and total scores were taken as missing.

The study also sought to understand whether changes were influenced by participant characteristics using linear regression modelling to determine the influence of explanatory variables. This enabled the effects of several possible explanatory variables to be evaluated together. The model was first fitted including using all participant characteristics to identify evidence of association. Participant characteristics for which there was no evidence of were removed and the model refitted (i.e., backward selection) (*p* < 0.05) to create the final model.

The participant characteristics included age at baseline, sex, ethnicity, employment status at 6 months post-stroke/TIA, living situation, and deprivation decile. We calculated deprivation deciles using the Index of Multiple Deprivation (IMD) for England. The IMD consists of a series of indices referring to seven domains of life. Decile 1 represents the most deprived area, and decile 10 the least deprived. In our model it was treated as a scale from 1 to 10, so that higher values indicated a lower degree of deprivation and, hence, a higher socioeconomic status. Our model indicates the effect of moving up one decile (i.e., to an area of lesser deprivation or greater socioeconomic status).

### 2.8. Patient and Public Involvement

No patient or public advisers were involved in setting the research question, outcome measures, or in the design and implementation of this study.

## 3. Results

**Figure 1:**
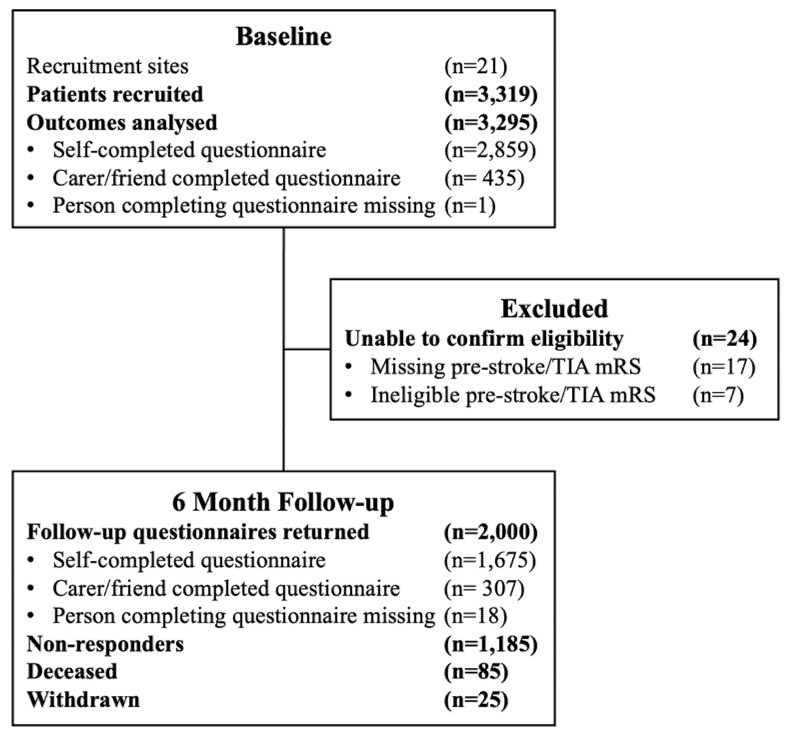
Participant recruitment and exclusion.

Twenty-one sites recruited 3295 eligible participants between December 2017 and April 2019. At baseline, 2859 (87%) questionnaires were self-completed; 435 (13%) were completed by a consultee; and one participant did not indicate who completed the questionnaire. There were 2000 6-month follow-up questionnaires returned (61% response rate); of which 1675 (85%) were self-completed and 307 (15%) were completed by a consultee; a further 18 responders did not indicate if the questionnaire was self-completed or not. By 6-month follow-up, 85 participants (3%) were deceased, 25 (1%) had withdrawn from the study, and 1185 participants (36%) were lost to follow-up (see Figure 1).

### 3.1. Participant Characteristics

Overall, participants’ baseline demographic characteristics were similar for all participants and the subset responding at 6-months (Table 1). There were, however, some notable changes including: an increase (4%) in the proportion of participants who got ‘out and about’ in their own car (though this question did not ask if it was participants that were driving the car); a change was also observed in the proportion of participants who were retired, increasing by (5%); and there was a small reduction (4%) in the proportion of participants from the lowest deprivation quintile.

When comparing the 2000 participants analysed at baseline and 6-months a number of changes in the demographics are noted over the 6-month period: a large decrease (20%) in the proportion of participants using their own vehicle, and an increase (13%) in reliance on friends/family members to “get out and about”; a decrease (7%) in the proportion of participants in full-time work; an increase (5%) in the proportion of participants unable to work; an increase (4%) in the proportion of participants who were retired.

Table 2 shows that participants analysed at both time points had a higher proportion of those reporting no symptoms on the pre-stroke/TIA mRS at baseline, than those initially recruited, by about 3%. It also shows that, of the participants who were analysed at both time points, there was an increased proportion of participants who required help with ADLs (20%) and any sensory problem (22%) from baseline to 6-months.

### 3.2. Non-Responders

**Figure 2:**
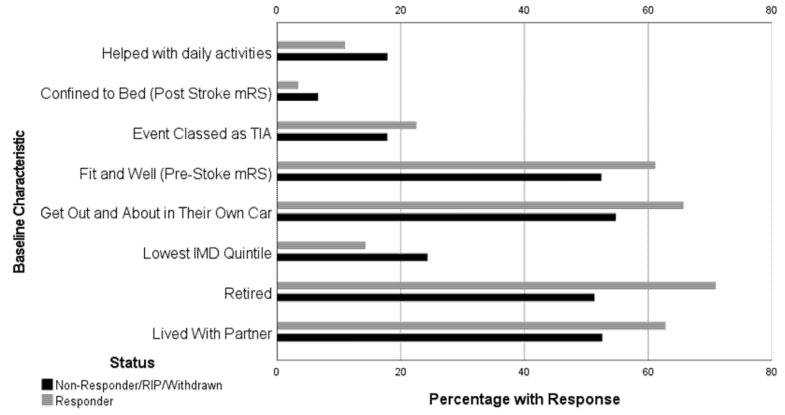
Differences in responders and non-responders baseline characteristics.

At 6-months, there were 2000 (61%) participant responses to the follow-up survey; 85 (3%) baseline participants had since died, 25 (1%) withdrawn, and 1185 (36%) were non-responders. Figure 2 shows that non-responders were less likely to be classed as having a TIA; were symptom free (pre-stroke/TIA mRS); got ‘out and about’ in their own car; were retired; or lived with a partner, when compared to responders. Meanwhile, a higher proportion of non-responders reported requiring help with daily activities; were confined to bed (post-stroke mRS); and were from the lowest deprivation quintile, when compared to responders. In addition, non-responders were on average younger (M = 70) than responders (M = 73).

### 3.3. Changes in Leisure Participation

**Figure 3:**
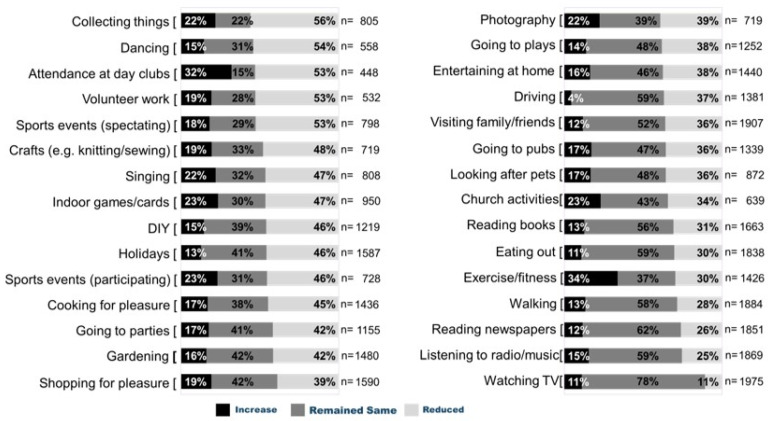
Changes in leisure participation between baseline and 6 months (ordered by highest proportion of reduction in participation).

Figure 3 shows the proportion of participants that reported an increase, reduction, or no change to each individual item of the sNLQ from baseline to 6-months excluding activities that remained as “never” engaged. Only one item, “exercise/fitness”, saw an increase (4%) in engagement from baseline to follow-up. There was no significant change in time spent “Watching TV” from baseline to follow-up. All remaining items on the sNLQ saw a reduction in participation between 10% and 40%, with a mean activity engagement reduction of 22%. Participants reported engaging in a mean of 16.5 leisure activities at baseline and 14.2 at 6-month follow-up.

Figure 3 shows that the most reduced activities were those which involved being physically active, such as dancing, or those requiring physical skill like knitting or singing. The activities that showed a higher proportion remaining the same were the more passive and sedentary, watching television, in particular, but also indoor activities such as looking after pets.

Table 3 shows the difference in the sNLQ total scores (level of activity), of the participants analysed at baseline and 6-months. Using the primary sNLQ assumption, 1988 of the potential 2000 results had responses to the sNLQ under the primary sNLQ calculation assumption to be compared in the paired *t*-test and gave a reduction in the sNLQ score of 5, 95% CI = [4.5, 5.2], (*p* < 0.001). There was a change in the mean differences in sNLQ total scores when considering all returned responses and the paired responses used in the *t*-test of 1; this suggests that participants with a lower sNLQ total score at baseline were less likely to return a 6-month questionnaire.

Table 4 shows the difference in the sNLQ count scores (variety of activity) of participants analysed at baseline to 6-months. The primary assumption allowed 1988 of the potential 2000 results to be compared in the paired *t*-test and gave a reduction in the sNLQ count score of 2, with 95% CI = [2.0, 2.4], (*p* < 0.001). There was a difference in the mean differences in sNLQ count scores when considering all returned responses and the paired responses used in the *t*-test of 0.5, suggesting that participants with a lower sNLQ count score at baseline were less likely to return a 6-month questionnaire.

There did not appear to be selection bias between responders and non-responders as mean scores in Table 3 and Table 4 indicate. Sensitivity analysis for the paired t-tests were conducted using an alternative assumption, that missing items on the NLQ caused the NLQ scores to be incalculable, this allowed 1334 of the potential 2000 participants to be included for use in the t-tests. With this assumption, a reduction in sNLQ total and count scores of 5, 95% CI = [4.5, 5.3], (*p* < 0.001) and 2, 95% CI = [1.9, 2.5], (*p* < 0.001). These results were like those under the primary assumption.

A model was first constructed with all the variables that might influence the response (Table 5). The variables that demonstrated weakest evidence of effect were removed. The variables with greatest evidence of effect were age at baseline, sex, and deprivation decile. We had no reason to suspect collinearity.

Table 6 presents the results of analysis of the influence of three factors on the change in the activity participation score (modelled as a ratio): age, sex (whether female), and socioeconomic indicated by deprivation decile.

The coefficients in the second column in this table indicate the respective effects of being a year older, female or living in an area one deprivation decile higher (i.e., less deprived). Thus, older age was associated with a reduction in the leisure activity score of about 1% for every additional 3 years of age. For female participants, leisure activity was 5% lower than for males. Living in an area one deprivation decile higher (i.e., to a less deprived area) was associated with an increase in leisure activities of 1%.

The confidence intervals provide a plausible range of values in the population from which the sample came. In all the variables in Table 6, both ends of the confidence interval were completely to one side or the other of 1, indicating evidence of an effect. However, the ends of the intervals were near 1, so that the actual effect might not be very large.

Overall, data showed that being older, female, or from a more deprived area, was associated with reduced engagement in leisure activity in 6-month period following stroke/TIA. According to our model, being in an upper deprivation decile would mean an increase in activity (i.e., a coefficient slightly above 1), whereas moving to a lower decile would mean a corresponding proportionate decrease.

## 4. Discussion

This is the largest national survey of changes in leisure practices following stroke/TIA in the world. Adoption of a quantitative survey method in collaboration with on-site research practitioners enabled the study to recruit from 21 sites across England, Wales, and Northern Ireland and achieve the intended sample size.

This study indicates that overall participation in leisure activities declined post-stroke/TIA, a finding which aligns with previous small-scale studies [8,9,30]; except for “exercise/fitness” which increased by 4% from baseline to follow-up. Engagement in exercise/fitness activities often declines following stroke [31,32] despite evidence that cardiorespiratory training and, albeit to a lesser extent mixed training (i.e., cardiorespiratory and resistance training), have been shown to reduce disability during or following usual stroke care [31]. However, these data provide only a snapshot of participants’ behaviour, and we do not have contextual information to further understand these trends in leisure engagement. For example, a patient might be completing increased exercise/fitness activities as part of their rehabilitation programme and so their belief is that their exercise/fitness engagement has increased, albeit for rehabilitation purposes. There are also further complications associated with the sNLQ as a measure of leisure participation—for example, a rehabilitation programme might have a focus on increasing walking activity leading a participant to report either walking, exercise/fitness, or both in their responses to the sNLQ—if the latter, participation would be double counted and not suitably representative of an individuals’ leisure activity engagement.

Watching television saw no overall change in engagement, which may suggest an already sedentary sample, or might indicate a lack of sensitivity associated with sNLQ. Whilst there was no overall change, the sNLQ does not ask participants to report the time spent involved in each activity. Previous studies [33] have highlighted that time spent sitting increases post-stroke and participants accumulated most of their sitting time whilst watching television whilst also exhibiting lower levels of physical activity compared to an aged-matched control group. Therefore, no overall change in engagement with this activity might be expected; however, understanding the time spent engaged in this activity would be beneficial to further exploring its potential impact upon rehabilitation, health, and wellbeing.

Our data showed that there was a 20% reduction in “getting out and about” in participants’ own vehicles from baseline to 6-months. Previous studies that have sought to specifically understand transportation choices following stroke/TIA have suggested that individuals wish to travel for specific purposes but also for its own sake (i.e., leisure) [34,35]. Furthermore, a longitudinal study of 145 Swedish stroke survivors [36] found that the ability to drive a car was a significant predictor of engagement in social and leisure activities at 10-year follow-up. Thus, clinicians should consider participants’ most common mode of transport as a mechanism to understand in order to better support individuals to engage in leisure activities.

Willingness to travel by driving, public transport, or walking, has been shown to be closely associated with an individual’s emotional state and can be adversely affected by gatekeeping from therapists, family members, and general practitioners [35,37]. As such, greater understanding of the psychological implications of travel, by any means, following stroke/TIA is required, particularly for leisure purposes. This has been further highlighted in recent studies that have explored the use of new technology, such as electrically assisted biked (e-bikes), which might be able to positively support and enable active travel, return to valued activities, and support rehabilitation [38].

Engagement in leisure activities has been shown to be important, with Mayo et al. [32] reporting that, 72% of participants (*n* = 434) lacked a valued or meaningful activity to occupy their time following a stroke, with leisure recognised as being important to support positive mental wellbeing, functioning, health status, and overall quality of life. In the present study, except for exercise/fitness and watching television, all of the remaining items on the sNLQ reduced between 10% and 40%. This included a range of physically active (i.e., dancing −40%) and sedentary (i.e., collecting things −34%). This suggests that in almost two decades, since Mayo and colleagues’ study, despite the increased recognition of the role that valued activities play in the long-term rehabilitation process [15], there remain unmet needs for leisure opportunities for individuals following stroke/TIA. Furthermore, clear emphasis is offered in the National Clinical Guidelines for Stroke, which advises practitioners to identify activities that people with stroke wish to participate in, help them overcome any associated barriers to participation, and provide information and referral to organisations that can support participation [39]. Likewise, the National Institute for Health and Care Excellence (NICE) Stroke Rehabilitation guidelines recommend that practitioners provide information about local resources that can help to support the leisure needs and priorities of the person with stroke and their family or carer [4].

There was a significant reduction (*p* < 0.001) in the number and variety of leisure activities that participants were engaging in at 6-month follow-up, and when testing for explanatory variables, age at baseline, sex, and socio-economic status were found to be significantly associated with leisure-based activity engagement.

Sex differences in stroke incidence, presentation, prevention, and treatment effectiveness are not fully understood [40]. A recent meta-analysis concluded that functional outcomes and quality of life after stroke are consistently poorer amongst women, even when controlling for baseline differences in age, pre-stroke mobility/function, and comorbidities [41]. Our data demonstrated that significant sex differences exist in terms of leisure participation following stroke/TIA and, thus, an additional factor broadly related to quality of life.

The evidence regarding outcome after stroke amongst different age ranges is better developed. Older individuals in our study were less likely to re-engage with leisure-based activities post-stroke and there are several factors that might have affected this: Firstly, older people are shown to receive a different pattern of clinical care; they tend to have a longer initial stay in hospital post-stroke, and require longer-term care once discharged [42]. Older adults’ declining engagement in leisure activities is not surprising as participation generally decreases with age [43]; though due to the sedentary living practices stroke survivors engage in post-stroke, we contend that it is imperative that rehabilitation includes high levels of active leisure, including resistance and aerobic exercise and that age and/or impairment should not be a barrier to engagement in these activities.

Socio-economic status is a well-established consideration for post-stroke screening as it is associated with incidence and post-stroke recovery [44,45,46]. A meta-analysis reported that low SES individuals are 1.7 times more likely to have a stroke than high SES individuals [46]; and that low SES stroke survivors are 1.7 times more likely to experience long-lasting disabilities than high SES individuals [47]. SES has been correlated to several inter-related indicators, income, education, and occupational status, though each indicator represents a distinctive element of SES that is uniquely related to health [48]. Our findings add an additional construct to the SES-health nexus, indicating that returning to leisure activities reduces following stroke, but there is a greater (significant) reduction amongst those with a low SES. There remains a need for more and better designed research on this issue [13], particularly when there is a growing body of related literature espousing the benefits of outdoor mobility and leisure-based activities [23,24,25].

Within our study, the most concerning factor, perhaps, is the convergence of the three explanatory variables; meaning that older females with a lower SES are the most likely to be negatively affected following stroke/TIA.

### Strengths and Limitations

To our knowledge, this is the largest study of its kind to examine changes in post-stroke/TIA leisure activity engagement utilising a well-established and pre-validated tool to do so. While we achieved a wide geographic spread, large sample and high questionnaire completion and return rates, the depth of understanding we have been able to achieve is limited by the research design. In particular, the sNLQ allowed us to measure overall changes in leisure participation, but not to explore the nature of those leisure activities. Thus, it is not possible to categorise types of leisure activities given the lack of definition/description for each activity within the sNLQ. One example might be photography; the overall sNLQ score may not indicate any change for an individual, but the nature of how they participate in this activity could possibly have altered dramatically (a move to photography within the home rather than outdoors, for instance). Furthermore, leisure activity engagement was self-reported, allowing potential misclassification or issues with item interpretation. This is an issue which should be addressed in future research to enable a fuller understanding of change. Similarly, issues with the mRS grading are well described in the literature; thus, matters of interobserver variability must be taken into consideration [49], particularly given the range and breadth of recruitment sites. There is also a likelihood of bias in respondents, as data highlighted that non-responders to the 6-month follow-up were less likely to live with a partner, less likely to have had a TIA, less likely to be “fit and well” (pre-stroke/TIA mRS), less likely to “get out and about in their own car”, less likely to be retired at baseline, and were likely to have lower sNLQ score at baseline than those who responded. Finally, a later or additional follow-up(s) (i.e., 12 months) might have provided a better indication of the lasting implications of participants’ stroke/TIA upon their life and leisure activities.

## 5. Conclusions

This study sought to examine changes in participation in usual life and leisure activities post- versus pre-stroke/TIA. Data demonstrated that there was an overall decline in the number of activities participants engaged in; averaging a drop of 2.2 activities, and an average loss of 4.9 in the level of activity undertaken following stroke/TIA. The only exceptions to this trend were participation in fitness/exercise which saw a small increase, and watching TV, which remained stable. There were significant reductions in activity participation observed amongst older participants, female participants, and those with a low SES. This study presents findings regarding health inequalities experienced by stroke survivors who are older, female, and from lower SES. We suggest that, due to the universal benefits that have been ascribed to being physically active across the lifespan, future intervention work should specifically ensure that they include approaches to engage these three participants groups post-stroke/TIA in physical activity(ies). However, in developing these interventions, further work is required to better understand the types of activities that these participant groups would like to engage in and the most appropriate time post-stroke/TIA for encouraging those discussions.

## Figures and Tables

**Table 1:** Baseline characteristics and demographics for participants analysed at baseline, 6-months and the 6-month responses.

Baseline Characteristics					Follow-Up Characteristics (*n* = 2000)	
Recruited at Baseline (*n* = 3295)		Responded at 6-Months * (*n* = 2000)				
				**Event, *n* (%)**		
2613	(79.3)	1549	(77.5)	Stroke	N/A	N/A
682	(20.7)	451	(22.6)	TIA	N/A	N/A
				**Age, median (IQR)**		
72.0	(61.0, 79.0)	73.0	(65.0, 80.0)		N/A	N/A
				**Sex, *n* (%)**		
1388	(42.1)	833	(41.7)	Female	N/A	N/A
1907	(57.9)	1167	(58.3)	Male	N/A	N/A
				**Ethnicity, *n* (%)**		
3159	(95.9)	1941	(97.1)	White	N/A	N/A
57	(1.7)	24	(1.2)	Asian	N/A	N/A
39	(1.2)	16	(0.8)	Black	N/A	N/A
14	(0.4)	7	(0.4)	Mixed	N/A	N/A
25	(0.8)	11	(0.6)	Other	N/A	N/A
				**Living situation, *n* (%)**		
1012	(30.8)	592	(29.6)	Alone	593	(29.9)
1935	(58.8)	1255	(62.8)	Partner	1201	(60.5)
311	(9.5)	141	(7.1)	Relative/Friend	142	(7.2)
33	(1.0)	10	(0.5)	Other	49	(2.5)
				**Get out and about, *n* (%)**		
2021	(61.4)	1312	(65.7)	In Their Own Car	912	(46.2)
551	(16.7)	314	(15.7)	Public Transport	318	(16.1)
146	(4.4)	59	(3.0)	Taxi	105	(5.3)
468	(14.2)	257	(12.9)	Relative/Friend	508	(25.7)
105	(3.2)	55	(2.8)	Other	133	(6.7)
				**Living situation, *n* (%)**		
3034	(92.2)	1887	(94.4)	Own Home	1824	(92.0)
145	(4.4)	57	(2.9)	Relatives Home	59	(3.0)
2	(0.1)	0	(0.0)	Care Home	36	(1.8)
76	(2.3)	36	(1.8)	Supported Living	37	(1.9)
35	(1.1)	18	(0.9)	Other	26	(1.3)
				**Employment, *n* (%)**		
610	(18.5)	334	(16.7)	Full-Time Work	190	(9.6)
220	(6.7)	128	(6.4)	Part-Time Work	98	(4.9)
41	(1.2)	14	(0.7)	Seeking Employment	14	(0.7)
139	(4.2)	48	(2.4)	Unable to Work	146	(7.4)
2176	(66.1)	1417	(70.9)	Retired	1486	(74.9)
106	(3.2)	57	(2.9)	Other	50	(2.5)
				**Socio-economic status (Quintiles), *n* (%)**		
601	(18.2)	286	(14.3)	1st (Most deprived)	N/A	N/A
649	(19.7)	353	(17.7)	2nd	N/A	N/A
682	(20.7)	445	(22.3)	3rd	N/A	N/A
683	(20.7)	448	(22.4)	4th	N/A	N/A
679	(20.6)	467	(23.4)	5th (Least deprived)	N/A	N/A

* Baseline information for participants that returned the 6-month questionnaire. “N/A” Refers to question not asked at 6-months.

**Table 2:** Baseline health status for participants analysed at baseline, 6-months and 6-month responses.

Baseline Characteristics						
Recruited at Baseline (*n* = 3295)		Responded at 6-Months * (*n* = 2000)			Follow-Up Characteristics (*n* = 2000)	
				**Pre-stroke mRS, *n* (%)**		
1902	(57.7)	1223	(61.2)	No Symptoms	N/A	N/A
1033	(31.4)	610	(30.5)	No Significant Disability	N/A	N/A
182	(5.5)	93	(4.7)	Slight Disability	N/A	N/A
178	(5.4)	74	(3.7)	Moderate Disability	N/A	N/A
				**Baseline mRS, *n* (%)**		
548	(16.6)	367	(18.4)	No Symptoms	530	(26.9)
1050	(31.9)	660	(33.0)	No Significant Disability	681	(34.5)
484	(14.7)	305	(15.3)	Slight Disability	256	(13.0)
434	(13.2)	243	(12.2)	Moderate Disability	313	(15.9)
624	(18.9)	356	(17.8)	Moderately Severe Disability	176	(8.9)
155	(4.7)	69	(3.5)	Severe Disability	16	(0.8)
				**Help with ADL, *n* (%)**		
443	(13.7)	216	(11.0)	Yes	611	(30.9)
				**Problems Reading, *n* (%)**		
190	(5.9)	103	(5.2)	Yes	296	(16.6)
				**Problems Writing, *n* (%)**		
200	(6.2)	103	(5.3)	Yes	416	(23.4)
				**Problems Speaking, *n* (%)**		
77	(2.4)	43	(2.2)	Yes	299	(17.0)
				**Problems Vision, *n* (%)**		
496	(15.2)	283	(14.3)	Yes	423	(23.7)
				**Problems Hearing, *n* (%)**		
548	(16.9)	332	(16.8)	Yes	432	(24.1)
				**Problems Language, *n* (%)**		
66	(2.1)	39	(2.0)	Yes	96	(5.8)
				**Problems Any, *n* (%)**		
976	(29.7)	573	(28.7)	Yes	991	(50.4)

* Baseline information for participants that returned the 6-month questionnaire. “N/A” Refers to question not asked at 6-months.

**Table 3:** sNLQ total scores from baseline to 6-months **.

	n	Mean	SD	Min	Q1	Median	Q3	Max	*p* Value
Baseline	1999	24.6	8.02	2.0	19.0	25.0	30.0	54.0	
6-Months	1989	19.7	8.53	0.0	13.0	20.0	26.0	47.0	<0.001 *

n, number; SD, Standard deviation; Min, minimum; Q1, Quartile 1; Q3, Quartile 3; Max, Maximum; *p* value, probability value. * Paired *t*-test Mean difference 4.9 with 95% CI = (4.52, 5.21) 1988 observations were used for the test. ** With the primary assumption that if not all activities are left blank, that a blank response means never participated.

**Table 4:** sNLQ count scores from baseline to 6-months **.

	n	Mean	SD	Min	Q1	Median	Q3	Max	*p* value
Baseline	1999	16.5	5.04	1.0	13.0	17.0	20.0	30.0	
6-Months	1989	14.2	5.76	0.0	10.0	14.0	18.0	29.0	<0.001 *

* Paired *t*-test Mean difference 2.2 with 95% CI = [1.99, 2.44]. 1988 observations were used for the test. ** With the primary assumption that if not all activities are left blank, that a blank response means never participated.

**Table 5:** Effect of participant characteristics (from modelling, before selection).

Explanatory Variable	Exponentiated Coefficient	*p* Value	95% Confidence Interval	
			Lower Limit	Upper Limit
Age at baseline	0.997	0.003	0.996	0.999
Female	0.953	0.025	0.913	0.994
Deprivation decile	1.011	0.004	1.003	1.019
If ethnic minority	0.997	0.141	0.992	1.001
Employed at 6 months	0.573	1.001	0.998	1.003
Employed at baseline	1.014	0.644	0.954	1.078
Living where category	0.960	0.390	0.875	1.053

**Table 6:** Effect of age, sex, and deprivation decile (from modelling, after selection).

Explanatory Variable	Exponentiated Coefficient	*p* Value	95% Confidence Interval	
			Lower Limit	Upper Limit
Age at baseline	0.997	0.003	0.996	0.999
Female	0.953	0.025	0.913	0.994
Deprivation decile	1.011	0.004	1.003	1.019

## Data Availability

The data that support the findings of this study are available from the UCLanData repository (https://uclandata.uclan.ac.uk/).
